# A cross-sectional analysis of health literacy: patient- versus family doctor-reported and associations with self-efficacy and chronic disease

**DOI:** 10.1186/s12875-021-01527-4

**Published:** 2021-09-15

**Authors:** Stephanie Stock, Sibel Altin, Farah Nawabi, Daniele Civello, Arim Shukri, Marcus Redaèlli, Adrienne Alayli

**Affiliations:** 1 Institute for Health Economics and Clinical Epidemiology, University of Cologne, Faculty of Medicine and University Hospital Cologne, Gleueler Str. 176-178, 50924 Cologne, Germany; 2 AOK Rheinland/Hamburg, Hamburg, Germany

**Keywords:** Health literacy, General practice, Self-efficacy, Chronic disease, Multimorbidity, Sex/gender

## Abstract

**Background:**

Adequate health literacy (HL) levels contribute to good health outcomes and successful disease self-management in patients with chronic disease. Hence, it is essential that family doctors recognize patients with inadequate HL in need of additional support. This study had two aims: (1) to assess and compare patient self-reported versus family doctor-rated HL estimates, and (2) to explore associations between patient-reported HL, self-efficacy and chronic diseases.

**Methods:**

Participants in this cross-sectional survey were recruited through general practices in North Rhine-Westphalia, Germany. Patient self-reported HL was measured using the European Health Literacy Survey-16. Family doctor-rated HL was measured with an adapted version of this instrument. Using crosstabulations patient-reported and family doctor-rated HL estimates were compared for 346 patient-family doctor pairs. Associations between HL, self-efficacy and chronic disease were investigated using regression analyses.

**Results:**

Patient-reported and family doctor-rated HL estimates were concordant in 38% of all cases. On average family doctors rated their patients’ HL lower than patients rated their own HL. The lower average family doctor ratings were more pronounced when patients were older, male and had more than one chronic disease. Female family doctors rated HL of male patients lower than their male colleagues. Patient reported HL had a significant positive association with self-efficacy. Mediation analysis provided support that self-efficacy acts as mediator between HL and the number of chronic diseases.

**Conclusions:**

Our study findings indicate a significant discrepancy between patients’ self-reported HL and externally rated HL by family doctors. A more systematic utilization of HL screeners might help reduce this discrepancy. At the same time, consideration should be given to enhancing communication training for family doctors and addressing critical HL skills in patient education.

## Background

Health literacy (HL) describes the ability to access, understand, appraise and apply (health-) information to make informed decisions regarding healthcare [1]. Adequate health literacy is important to achieve good health outcomes in chronic care [2, 3]. Health literacy also emerges as predictor for inequalities in health, because patients with higher HL levels are more likely to utilize preventive health services and exhibit better disease self-management [4–8]. The latter is crucial for coping with chronic disease and for maintaining a high quality of life [9].

Family doctors in Germany serve a high proportion of chronically ill patients. Their ability to judge their patients’ HL adequately can help identify patients in need of additional support. Screening patients HL is not common in the general practice setting. Rather, family doctors often subconsciously use their patients’ education status, appearance, or manner of speaking as proxy [10].

Few studies investigate physicians’ ability to judge their patients’ HL [11–13]. Mechanisms involved in the relationship between HL and chronic disease outcomes are still poorly understood [14]. Several patient- and system-related factors have been proposed as mediators in the causal pathways linking health literacy and health outcomes [15]. Increasingly, research suggests patient self-efficacy as a mediator and hence as potential intervention target to positively affect chronic disease outcomes [16, 17]. Repeatedly, studies have demonstrated that self-efficacy is positively associated with HL [18] and that self-efficacy predicts disease self-management behaviors [19]. The evidence on pathways between HL, self efficacy and chronic disease outcomes is however limited to date [17, 20]. Evidence is also lacking regarding the relationship between HL and multimorbidity, the co-occurrence of two or more chronic diseases [21]. Insights into factors affecting a patients’ number of chronic diseases are necessary to better understand mechanisms involved in preventing and delaying multimorbidity.

This study assessed family doctors’ ratings of their patients’ HL compared to patient self-reported HL in a general practice setting. We also explored associations between patient reported HL, self-efficacy and the number of chronic diseases.

## Methods

Between October 2015 and December 2017 an invitation to participate in the cross-sectional survey was faxed to 208 general practices in the German state of North Rhine-Westphalia. A member of the research team visited practices who expressed an interest to participate (n = 11) to provide further information and enrol family doctors in the study. Patients were recruited by their family doctors during regular consultations. A member of the research team was present in the participating practices on previously scheduled dates to support the recruitment process and provide information to patients eligible for participation.

Patients were included if they were at least 18 years of age, spoke German, and had at least two consultations with their family doctor during the past 12 months.

### Measures

Patients’ self-reported HL was measured using the German version of the European Health literacy Survey HLS-EU-16 [22]. Family doctors rated their patients’ HL with an adapted version of the HLS-EU-16 in which questions were reframed so family doctors could answer as proxies for their patients. The version was pretested by the study team with family doctors in their practices.

The HLS-EU-16 is a 16-item short version of the HLS-EU built on a conceptual model of HL after an extensive literature review [22]. It measures four HL skills with respect to information processing (i.e. accessing, understanding, appraising, and applying health information) in three settings (i.e. healthcare, prevention, health promotion). On a scale ranging from “very easy” to “very difficult”, patients indicate how easy it is for them to perform different activities. The short version has acceptable psychometric properties, and its sum score correlates highly with the sum score of the long version (r = 0.82) [23]. Following the scoring system suggested by the developers of the instrument all items were dichotmised by collapsing the two adjoining categories and a simple sum score across the 16 binary items was calculated. Based on the sum score three levels of health literacy were distinguished, using the same cut-off values as other international versions: inadequate (score ≤ 8), problematic (score > 8 and ≤ 12), and adequate (score > 12). Only respondents with at least 14 valid answers were included in further analyses [23].

We used a general measure of self-efficacy to measure patients’ perception of their ability to perform across a variety of different situations [24]. The general self-efficacy short form (Allgemeine Selbstwirksamkeit Kurzskala, ASKU) [25] is a validated short instrument with 3 items, scored on a 5-point Likert scale (Table 1). For the analysis, the mean of each item is calculated and compared with a reference table. Psychometric properties are acceptable (r = 0.86) [25].

**Table 1:** Items of the ASKU

Items^a^	do not agree at all	hardly agree	somewhat agree	mostly agree	completely agree
1. *I can rely on my own abilities in difficult situations*	□	□	□	□	□
2. *I am able to solve most problems on my own*	□	□	□	□	□
3. *I can usually solve even challenging and complex tasks well*	□	□	□	□	□

^a^Items are derived from Beierlein et al. 2013 [25]. The authors of this article have translated the original items from German into English. An official translation into English is underway: Doll, E., Nießen, D., Schmidt, I., Rammstedt, B., & Lechner, C. M. (2020). The General Self-Efficacy Short Scale–3 (GSE-3): An English-language adaptation. Manuscript in preparation

### Data analyses

Demographic variables and HL questions were analysed using descriptive statistics. The patient sample was split into subgroups based on their HL levels (i.e. inadequate, problematic and adequate). In case of disagreement, a distinction was made into “moderate disagreement “ (difference by one level) and “marked disagreement “ (difference by two levels). Differences in mean scores between patient self-reported and family doctor rated HL were compared using t-tests. Multivariate linear regression analyses were conducted to investigate associations of patient-reported HL estimates with self-efficacy and chronic disease to assess for evidence of mediation. Results were considered significant in case of p < 0.05. All analyses were conducted using IBM® SPSS® Statistics for Windows, version 26.0 [26–32].

## Results

Eleven practices with twenty-eight family doctors were enrolled in the study. Completed surveys were returned from fourteen family doctors in eight practices. Characteristics of the family doctors and practices included in the survey are summarized in Table 2.

**Table 2:** Characteristics of participating family doctors and practices

Characteristic		Number	Percent
Family doctors (n=14)			
*Gender*	*Male*	*9*	*64.3*
	*Female*	*5*	*35.7*
Participating practices (n=8)			
*Number of patients* *Enrolled* ^*a*^	*≥500-1000*	*1*	*14.3*
	*>1000-1500*	*3*	*42.9*
	*>1500-2000*	*2*	*28.6*
	*>2000*	*1*	*14.3*
*Type*	*Single practice*	*3*	*37.5* *62.5*
	*Group practice*	*5*	
*Location*	*Metro*	*6*	*75.0*
	*Urban*	*2*	*25.0*

^a^Data for one practice is missing

The participating family doctors recruited 346 patients of whom 93.8% were chronically ill. Response rates to the patient survey were 96% and 100% to the family doctor survey respectively. Characteristics of the patient sample are displayed in Table 3.

**Table 3:** Characteristics of the patient sample

				Number	Percent
*Gender* ^*a*^	*Female*			*200*	*57.8*
	*Male*			*135*	*39.0*
*Age* ^*b*^	*≤ 60 years*			*164*	*47.4*
	*> 60 years*			*165*	*47.7*
*Employment status* ^*c*^	*Not working*			*166*	*48.0*
	*Working*			*166*	*48.0*
*Education level* ^*d*^	*Low*			*144*	*41.6*
	*Moderate*			*89*	*25.7*
	*High*			*102*	*29.5*
*Migration background* ^*e*^	*No Migration background*			*253*	*73.1*
	*With migration background*			*77*	*22.3*
*Number of chronic diseases* ^*f*^	*None*			*21*	*6.1*
	*1*			*101*	*29.2*
	*2*			*98*	*28.3*
	*3 or more*			*121*	*35.0*
*Chronic diseases* ^*f*^	*Cardiovascular disease*			*183*	*53.7*
	*Back pain*			*150*	*44.0*
	*Depression or other* *Mental disorders*			*84*	*24.6*
	*Diabetes*			*82*	*24.0*
	*Chronic obstructive pulmonary disease*			*56*	*16.4*
	*Cancer*			*36*	*10.6*
	*Rheumatism*			*35*	*10.3*
	*Stroke*			*16*	*4.7*
	*Chronic kidney disease*			*6*	*1.8*
	*Other*			*101*	*29.6*
	**n**	**Mean**	**SD**	**Min**	**Max**
*Age* ^*b*^	*329*	*57.9*	*16.4*	*19*	*89*
*ASKU Score* ^*g,*^ * [1=low to 5=high]	*341*	*3.9*	*0.8*	*1*	*5*
HLS-EU-16 Score** [0=low/no HL to 16=high HL]	*293*	*12.2*	*3.3*	*1*	*16*

*Missing data:* ^*a*^ *11,* ^*b*^ *17,* ^*c*^ *14,* ^*d*^ *11,* ^*e*^ *16,* ^*f*^ *5,* ^*g*^ *5*

*The ASKU score is calculated by taking the mean of the three items

**Patient self-reported, only includes respondents with at least 14 valid answers

The majority (52.9%) of patients surveyed rated their HL levels as adequate, 32.8% as problematic, and 14.3% as inadequate.

Stratified analyses of the HL sum score showed that patients with employment and high education level had significantly higher self-reported HL levels (p = 0.002; p = 0.005). Stratified analyses by age (p = 0.995), sex (p = 0.474) and migration (p = 0.640) background indicated no significant differences in self-reported HL.

### Patient-reported versus family doctor-rated health literacy

**Fig. 1:**
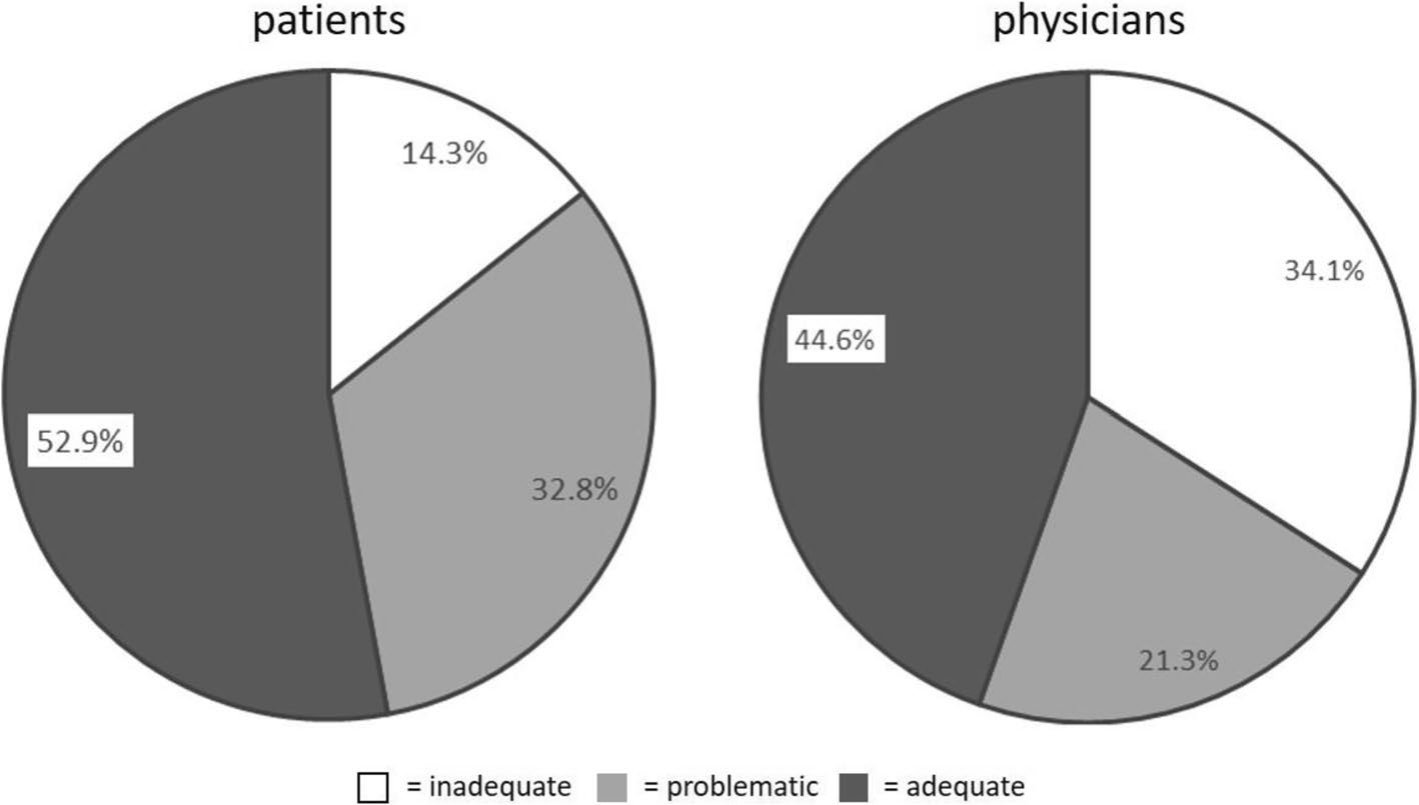
Patient-reported versus family doctors-rated HL levels (%)

Figure 1 shows proportions of the adequate, problematic and inadequate HL categories in patient self-reported and family doctor-rated HL estimates.

Patient-reported and family doctor-rated HL estimates were concordant in 38% of all cases. In 40.8% of the cases the estimates differed moderately, and in 21.1% markedly. Family doctors’ estimates of their patients’ HL were higher than patients’ self-assessment in 22.9% of cases. Family doctors’ estimates were lower than their patients’ HL estimates in 39.0% of the cases. Patient-reported and family doctor reported HL were most frequently concordant in the adequate HL category (26%) and least frequently concordant in the inadequate HL category (4%).

Mean difference scores (MDS) between patient-reported and family doctor-rated HL estimates are presented in Table 4. On average family doctors’ HL ratings were lower than their patients’ self-reported HL. Stratified analyses showed that the lower family doctor ratings were significantly more pronounced when patients were older, male and had more than one chronic disease.

The lower family doctor ratings were also more pronounced when female doctors carried out HL ratings. This difference was not significant, however (see Table 4). Further stratification by both patient sex and family doctor sex showed that female family doctors in our sample underestimated HL of male patients to a larger extent than their male colleagues (female family doctors: MDS = 3.41 for male vs. 0.51 for female patients, p = 0.042; male family doctors: MDS = 2.06 for male vs. 0.87 for female patients, p = 0.191).

**Table 4:** Comparison of Mean HL Scores

Full analysis		Mean difference score	p
*HLS-Score patient (12.44)*		*1.42*	*<0.001*
*HLS-Score family doctor (11.01)*			
**Stratified analyses**			
*Patient characteristics*			
*Age*	*> 60*	*2.75*	0.001**
	*≤ 60*	*0.23*	
*Gender*	*Male*	*2.38*	0.032*
	*Female*	*0.75*	
*Migration background*	*Yes*	*1.47*	*0.976*
	*No*	*1.50*	
*Number of chronic diseases*	*≤1*	*-0.01*	0.007**
	*>1*	*2.11*	
*Family doctor characteristics*			
*Gender*	*Male*	*1.38*	*0.849*
	*Female*	*1.53*	

*p<.05 **p<.01

### Associations of patient-reported health literacy with self-efficacy and chronic disease

Stepwise regression analyses showed that the explained variance in patients’ self-efficacy improved significantly by including the patients’ HL sum score as predictor in a model consisting of socio-demographic predictors only (see Table 5). In this model only education level and HL had a significant independent contribution to self-efficacy.

**Table 5:** Linear regression models

Independent variables	R^**2**^	R^**2**^ change	B	SE	p
*Model 1*	*0.078*	*0.078*			
*Gender*			*0.104*	*0.094*	*0.268*
*Age*			*0.163*	*0.104*	*0.119*
*Migration background*			*-0.149*	*0.106*	*0.161*
*Education level*			*0.186*	*0.056*	0.001**
*Employment status*			*0.239*	*0.101*	0.018*
Model 2	0.231	*0.153*			
*Gender*			*0.100*	*0.086*	*0.244*
*Age*			*0.070*	*0.096*	*0.469*
*Migration background*			*-0.121*	*0.097*	*0.214*
*Education level*			*0.112*	*0.053*	0.033*
*Employment status*			*0.103*	*0.094*	*0.273*
*HL*			*0.094*	*0.013*	0.000***

*p<.05 **p<.01 ***p<.001

dependent variable = ASKU score

Mediation analyses were conducted to test the hypothesis that patients’ self-efficacy mediates the relationship between HL and the number of chronic diseases. In line with this hypothesis, we found a significant positive association between HL and self-efficacy (beta = 0.102, p = 0.000) and a significant negative association between self-efficacy and number of chronic diseases (beta = -0.289, p = 0.005). The association between HL and chronic diseases was negative as expected, however did not reach significance (beta = -0.024, p = 0.317).

## Discussion

This study assessed concordance between patients’ self-reported and family doctor-estimated HL for chronically ill patients in a general practice setting. It also explored associations between patient-reported HL, self-efficacy and the number of chronic diseases.

The distribution of self-reported HL in our patient sample compares to the distribution for adults equal or above 60 years in the German general population and can be judged to be fairly representative [33]. International research indicates that a lower education and being male are associated with lower HL levels [34, 35]. This could only be reproduced for education in our sample. In contrast to previous general population data [36], participants with migration background did not have lower HL levels in our study. The relatively high education levels of migrants included in our study might be responsible for this finding. Compared to the average of the German population [37] patients in our sample more often had a higher school education or degree that qualified for university entrance.

Analyses of patient-reported versus family doctor-rated HL estimates showed that these were concordant in 38% of the cases. This limited concordance was also found by the few available international studies, which have shown an overestimation of patients’ HL by physicians in primary care and other health care providers [11–13, 38]. In contrast to these studies, the present survey shows that in a German general practice setting family doctors on average rate their patients’ HL lower than patients rate their own HL. A possible explanation could be the use of a more sophisticated instrument in this study.

This study identified several factors affecting the concordance between family doctors’ ratings and patients’ self-reported HL. Ratings were most frequently concordant in patients with adequate HL (26%) and least frequently concordant in patients with inadequate HL (4%). The lower average family doctor ratings found in our sample were more pronounced when patients were older, male and had more than one chronic disease.

The tendency of family doctors to provide lower HL-ratings for elderly patients (> 60 years of age) might be explained by the perception of a general decrease of HL with age as observed in the European HL Study [34] and US studies [39, 40].

The lower family doctor ratings for male patients might be attributed to the observation that men engage less in seeking health information, screening programs and health promoting behaviors than women [41–43]. This phenomenon is documented by an increasing body of research, yet an unterstanding of specific mechanisms leading to sex and gender-based differences in health behaviors are currently lacking [44, 45]. Interestingly, female family doctors in our study rated HL of male patients lower than their male colleagues. This difference suggests that male and female doctors may have different perceptions about health literacy of male and female patients. This finding is concordant with other studies observing differences in clinical judgements regarding male and female patients based on physicians’ sex [46].

Family doctors in our study rated their patients HL significantly lower when they had more than one chronic disease. This may suggest that family doctors make inferences about HL levels based on the assumption that multimorbidity is are determined by patients’ lifestyle choices. Evidence is emerging that unhealthy lifestyle choices, such as low levels of physical activity and smoking increase the likelihood of multimorbidity [47, 48]. A scientific basis for an association between HL and multimorbidity is currently lacking, but it is possible that this association exists for specific combinations of diseases [21] or specific HL profiles [49].

In line with previous studies investigating the relationship between HL and self-efficacy among patients with diabetes and heart failure [50–52], we found a significant positive association between HL and self-efficacy among chronically ill patients. Mediation analysis provided support that self-efficacy acts as mediator between HL and the number of chronic diseases.

### Strenghts and limitations

A strength of our study is the relatively large number of patient-family doctor pairs surveyed and the high response rate of patients and doctors in the participating practices. The use of an adapted version of the HLS-EU-16 to derive family doctor-rated HL estimates is another strength. Previous studies have relied on single-item tools, asking family doctors to directly assign patients to a certain health literacy level [11–13, 38]. A limitation is that we had to rely on self-reports for all outcomes and thus could not validate patients’ or family doctors’ HL estimation against an objective standard. Second, participation was voluntary for practices, family doctors and patients. Hence, we cannot preclude selection bias.

### Clinical implications

This study provides further evidence that family doctors have difficulties in rating their patients’ HL. The finding that family doctor HL ratings were significantly lower for older patients, male patients, and patients with more than one chronic disease suggests that family doctors make inferences about patients’ HL based on other variables. Additionally, we found that ratings of patients HL differed between male and female family doctors. Consequently, family doctors cannot optimally tailor interactions to their patients’ HL levels. This underscores the importance that family doctors either use objective tools to screen patients regarding their HL or use plain language in interactions with all patients. Both strategies have pros and cons. Xu et al. [53] conclude that clear explanations from physicians have the potential to improve patients’ HL (specifically communicative and critical HL) and self-efficacy. This may support the request that physicians should use plain language with all patients [53]. Seligman et al. [10] report that physicians respond to patients identified with low HL with changed communication patterns, but at the same time feel less satisfied with the visit. Other challenges with institutionalized screening programs relate to time barriers and shaming patients [54]. To enhance patient-centered communication physicians need training in applying screening tools and communication techniques for patients with low HL. Confirming whether patients understand the information provided (e.g., using the teach-back method [55]) and strategies to enhance patients’ self-efficacy are also essential steps in improving self-management education.

### Future research

Qualitative research is essential to understand why family doctors have difficulties judging their patients’ HL. It can also provide further insights into sex/gender-based differences in family doctor ratings. Existing training approaches for health professionals to adapt to their patients’ HL-levels are useful, but require further development and testing [56, 57]. Short HL screeners like the one from Chew et al. [58] show promising results. Further validation studies in different settings and populations are recommended to facilitate use of the screener in routine practice [58]. Future studies should also focus on refining alternative methods to estimate patient HL levels from routine primary care health records [59]. Finally, gender influences in patient-doctor interaction should be further investigated. Gender-sensitive approaches have been identified as an important factor in patient-centeredness of care, that need consideration in addition to patients HL levels [60].

## Conclusion

The results of this study show that there is a significant discrepancy between patients’ self-reported HL and external assessment of HL by their family doctors. It is possible that the systematic use of HL screeners by family doctors could help reduce this discrepancy. To avoid patient stigmatisation consideration should be given to incorporating communication training into all family physician training curricula and to addressing critical HL skills in traditional patient education.

## Data Availability

The datasets generated and/or analysed during the current study are not publicly available due to their containing information that could compromise the privacy of research participants. However, summarised data are available upon reasonable request.

## Abbreviations

HL: Health literacy
HLS-EU: European Health Literacy Survey
ASKU: Allgemeine Selbstwirksamkeit Kurzskala
